# Supplementary material for: Identification and classification of ion-channels across the tree of life provide functional insights into understudied CALHM channels
Source: bioRxiv. 2025 Sep 28:2025.02.10.637530. Preprint. [Version 3] doi: 10.1101/2025.02.10.637530 (PMC12485921; doi:10.1101/2025.02.10.637530)
Supplement: Supplement 1 [file NIHPP2025.02.10.637530v3-supplement-1.pdf]

bioRxiv preprint doi: https://doi.org/10.1101/2025.02.10.637530; this version posted September 28, 2025. The copyright holder for this preprint (which was not certified by peer review) is the author/funder, who has granted bioRxiv a license to display the preprint in perpetuity. It is made available under aCC-BY 4.0 International license.

Supplementary Figures

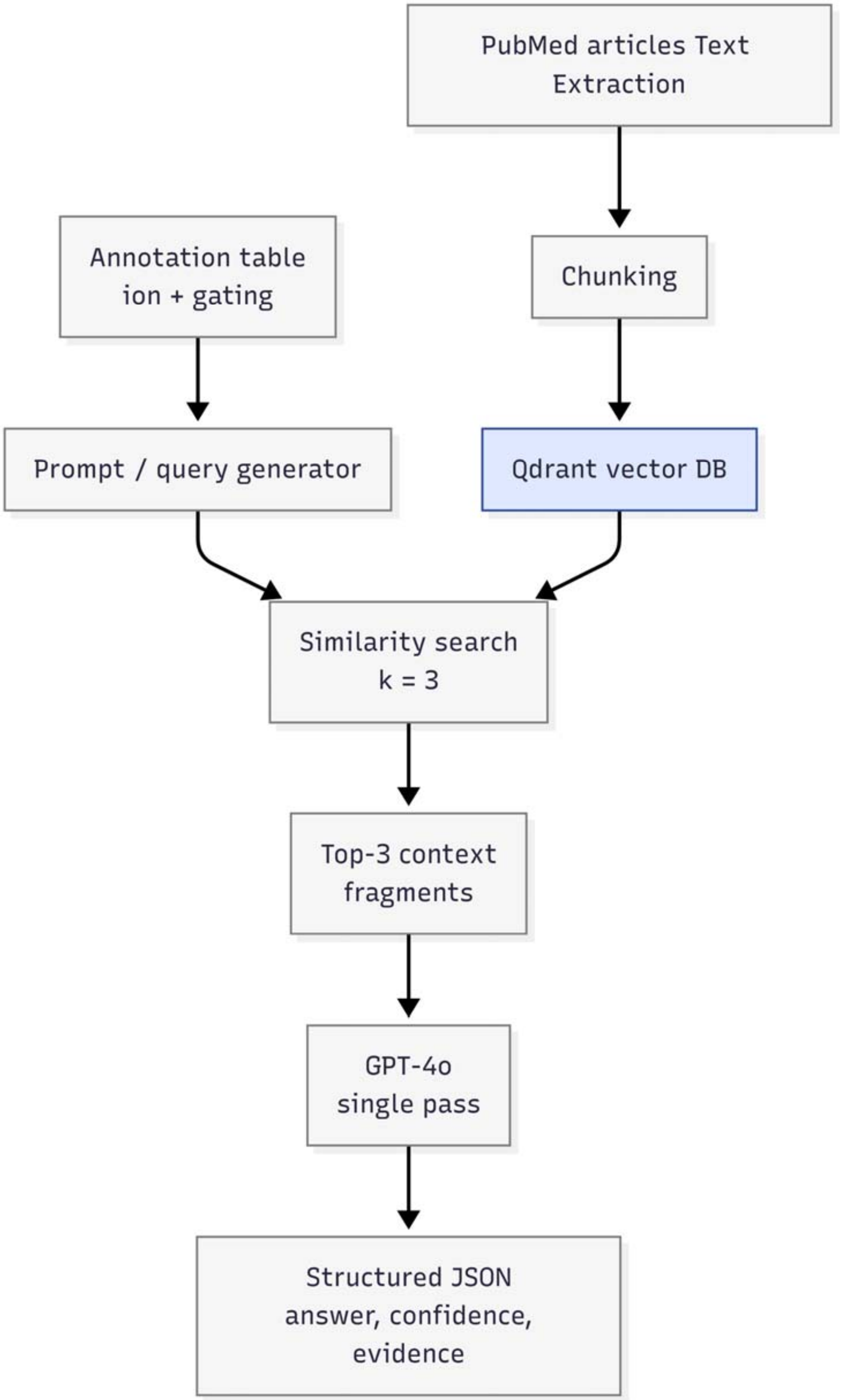

Supplementary Figure 1: RAG annotation pipeline used for validating the annotation of ion specificity and gating mechanism

bioRxiv preprint doi: https://doi.org/10.1101/2025.02.10.637530; this version posted September 28, 2025. The copyright holder for this preprint (which was not certified by peer review) is the author/funder, who has granted bioRxiv a license to display the preprint in perpetuity. It is made available under aCC-BY 4.0 International license.

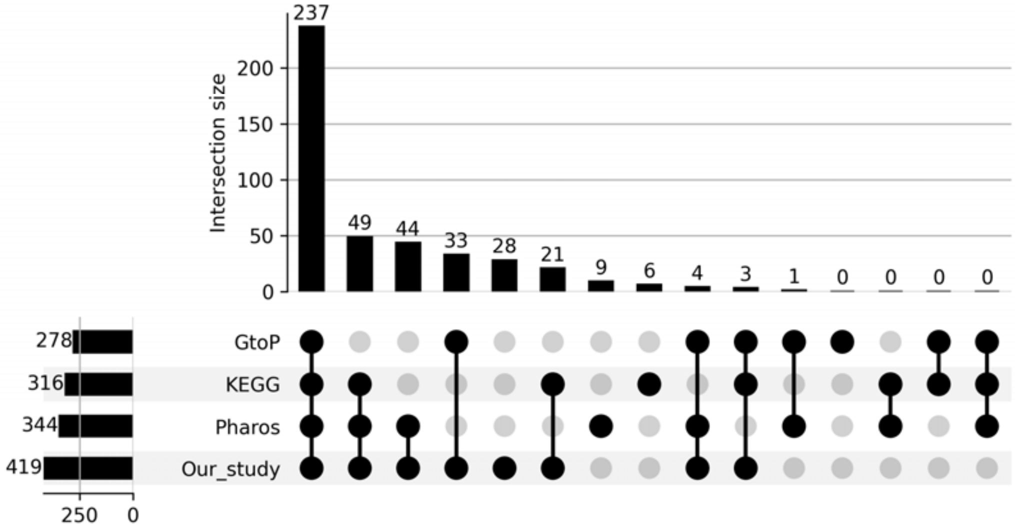

Supplementary Figure 2: Upset plot showing the overlap of IC sequences based on their UniProt IDs across the current study, the KEGG database, GtoP and Pharos.

bioRxiv preprint doi: https://doi.org/10.1101/2025.02.10.637530; this version posted September 28, 2025. The copyright holder for this preprint (which was not certified by peer review) is the author/funder, who has granted bioRxiv a license to display the preprint in perpetuity. It is made available under aCC-BY 4.0 International license.

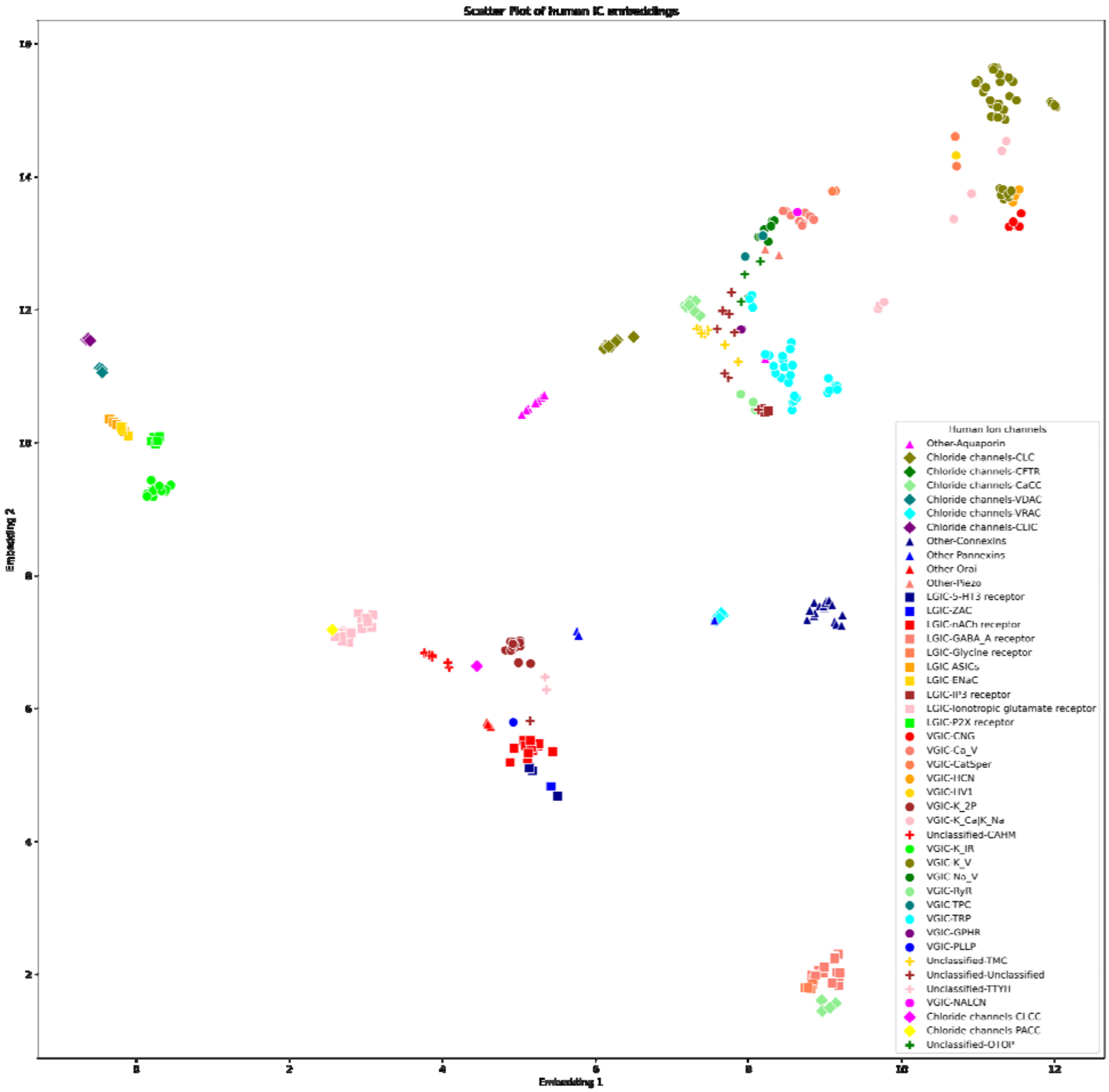

Supplementary Figure 3: UMAP embeddings plot of all human IC sequences. The UMAP embeddings were generated based on the pairwise similarity scores of the protein embedding alignment generated for all pairs of human ICs. Shapes of the markers indicate the different IC groups (circle: VGIC, square: LGIC, diamond: Chloride channel, triangle: Other, plus: Unclassified) and the colors indicate different IC families within each group as shown in the legend above.

bioRxiv preprint doi: https://doi.org/10.1101/2025.02.10.637530; this version posted September 28, 2025. The copyright holder for this preprint (which was not certified by peer review) is the author/funder, who has granted bioRxiv a license to display the preprint in perpetuity. It is made available under aCC-BY 4.0 International license.

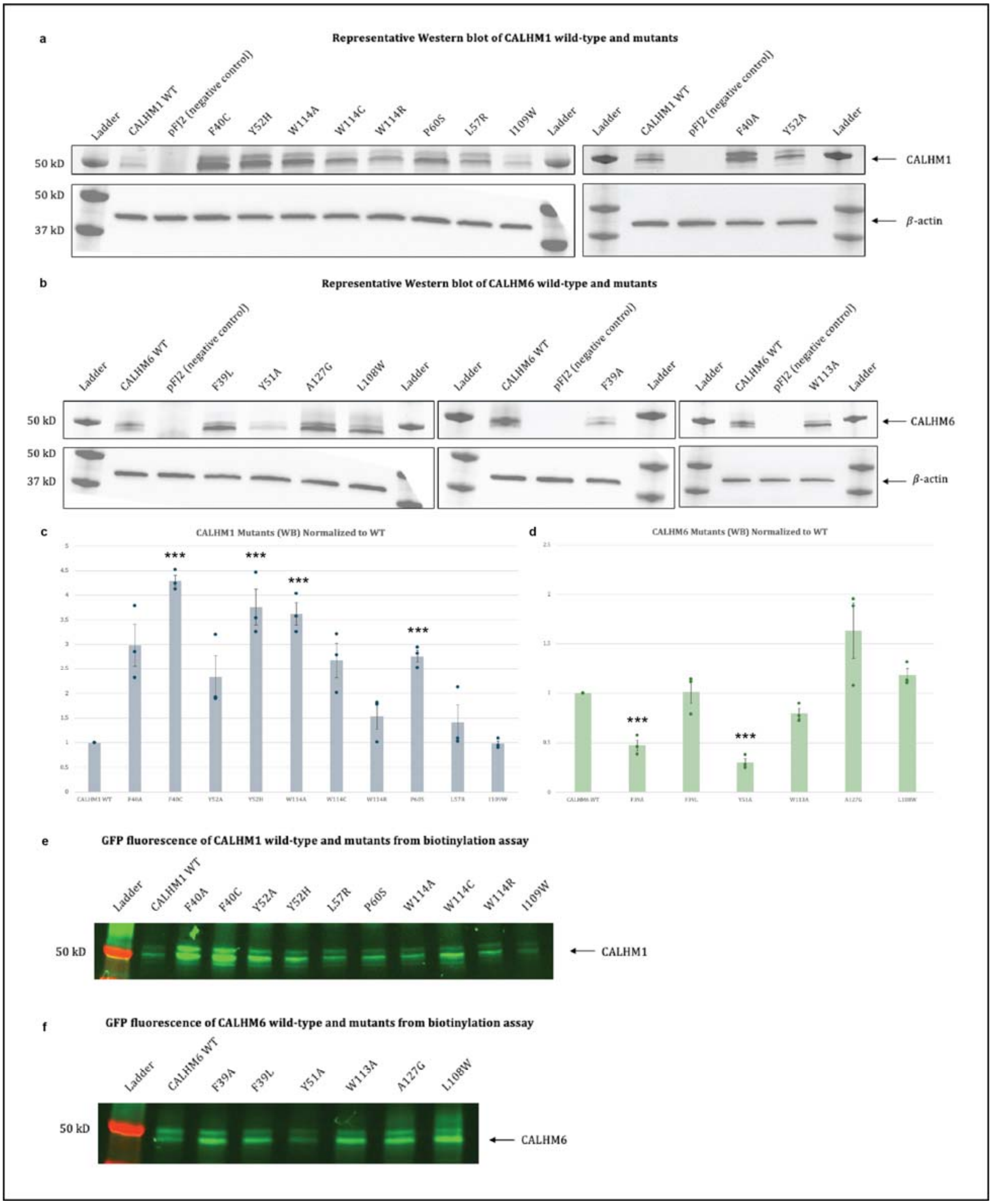

Supplementary Figure 4. Expression analysis of wild-type CALHM1, wild-type CALHM6, and their mutants. (A,B) Representative gels showing CALHM1 and its mutants (A), and CALHM6 and its mutants (B). CALHM1 and CALHM6 signals were detected using in-gel fluorescence of

bioRxiv preprint doi: https://doi.org/10.1101/2025.02.10.637530; this version posted September 28, 2025. The copyright holder for this preprint (which was not certified by peer review) is the author/funder, who has granted bioRxiv a license to display the preprint in perpetuity. It is made available under aCC-BY 4.0 International license.

the C-terminal GFP tag, while β-actin was detected by Western blotting as a loading control. (C,D) Quantification of total protein expression levels of wild-type CALHM1 and its mutants (C), and wild-type CALHM6 and its mutants (D). Each dot represents an independent measurement (transfection) and error bars represent SEM (c, n = 3; d, n = 3). Statistical analysis was performed using one-way ANOVA with Bonferroni's post hoc test, comparing each mutant to wild type (*$p < 0.05$; **$p < 0.01$; ***$p < 0.001$). (E,F) Surface biotinylation assays of wild-type CALHM1 and its mutants (E), and wild-type CALHM6 and its mutants (F), detected using the C-terminal GFP tag by in-gel fluorescence.

bioRxiv preprint doi: https://doi.org/10.1101/2025.02.10.637530; this version posted September 28, 2025. The copyright holder for this preprint (which was not certified by peer review) is the author/funder, who has granted bioRxiv a license to display the preprint in perpetuity. It is made available under aCC-BY 4.0 International license.

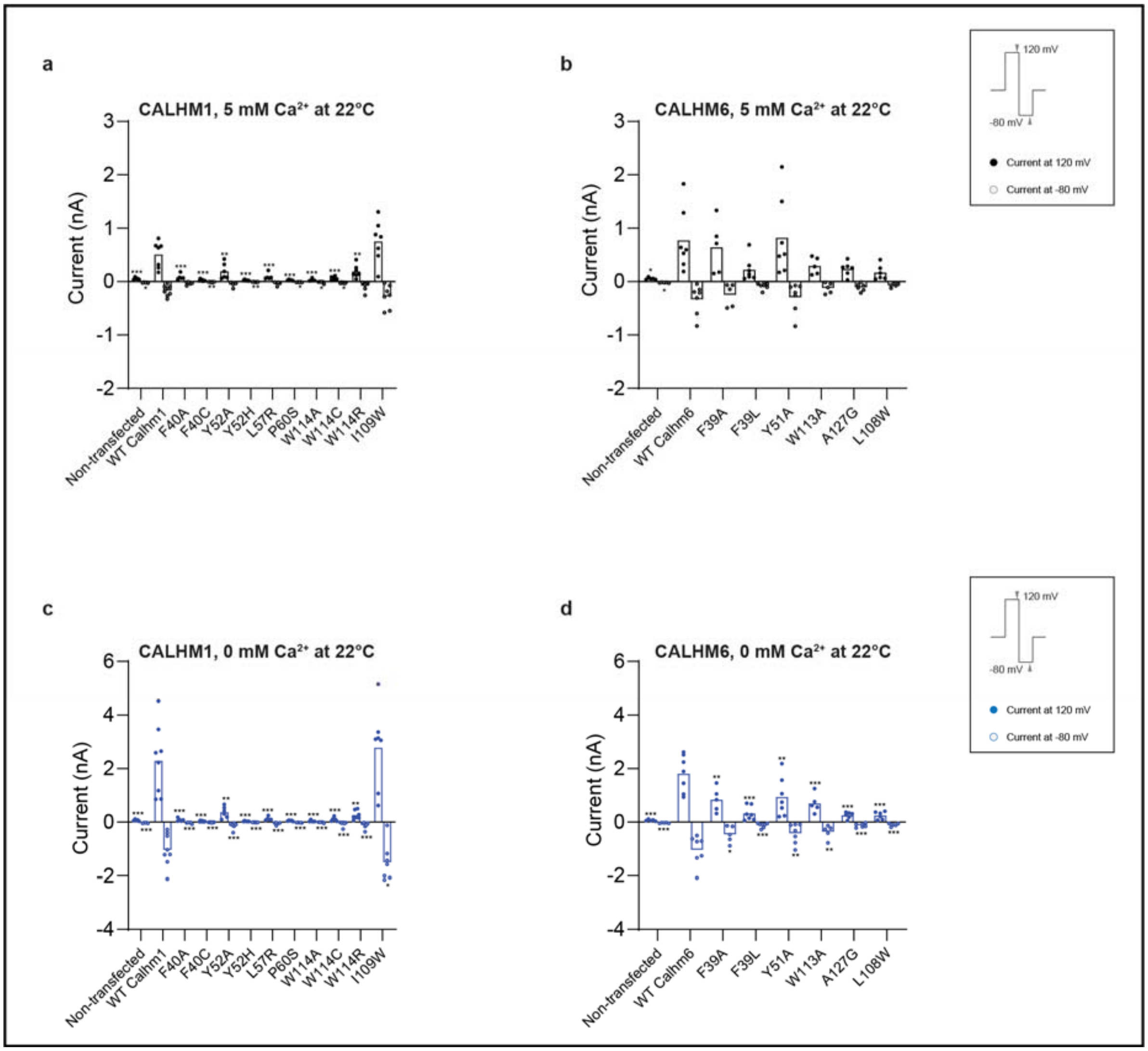

Supplementary Figure 5: Functional characterization of CALHM1 and CALHM6 mutants at conserved residues at 22 °C. Current amplitudes obtained using a two-step voltage protocol (from +120 mV to −80 mV; protocol shown in the box on the right) are compared between wild-type CALHM1 and its mutants (A, B) and between wild-type CALHM6 and its mutants (C, D). The cells analyzed here are the same as those in Figure 5P, Q. Briefly, for each cell, currents were measured sequentially under three conditions: 5 mM Ca^{2+} at 22 °C, 0 mM Ca^{2+} at 22 °C, and 0 mM Ca^{2+} at 37 °C. The currents from the first two conditions are plotted here, while currents at 0 mM Ca^{2+} at 37 °C are shown in Figure 5R, S. Each dot represents an independent measurement (cell), and bar represents the mean current amplitude across cells. The number of independent measurements (cells) for each bar in a–d are shown from left to right: 5, 8, 5, 8, 6, 7, 5, 5, 5, 6, 7, 7 (A, B); 5, 6, 5, 7, 7, 6, 6, 5 (C, D). Statistical analysis was performed using one-way ANOVA with Bonferroni's post hoc test, comparing each mutant to wild type (*$p < 0.05$; **$p < 0.01$; ***$p < 0.001$).

bioRxiv preprint doi: https://doi.org/10.1101/2025.02.10.637530; this version posted September 28, 2025. The copyright holder for this preprint (which was not certified by peer review) is the author/funder, who has granted bioRxiv a license to display the preprint in perpetuity. It is made available under aCC-BY 4.0 International license.

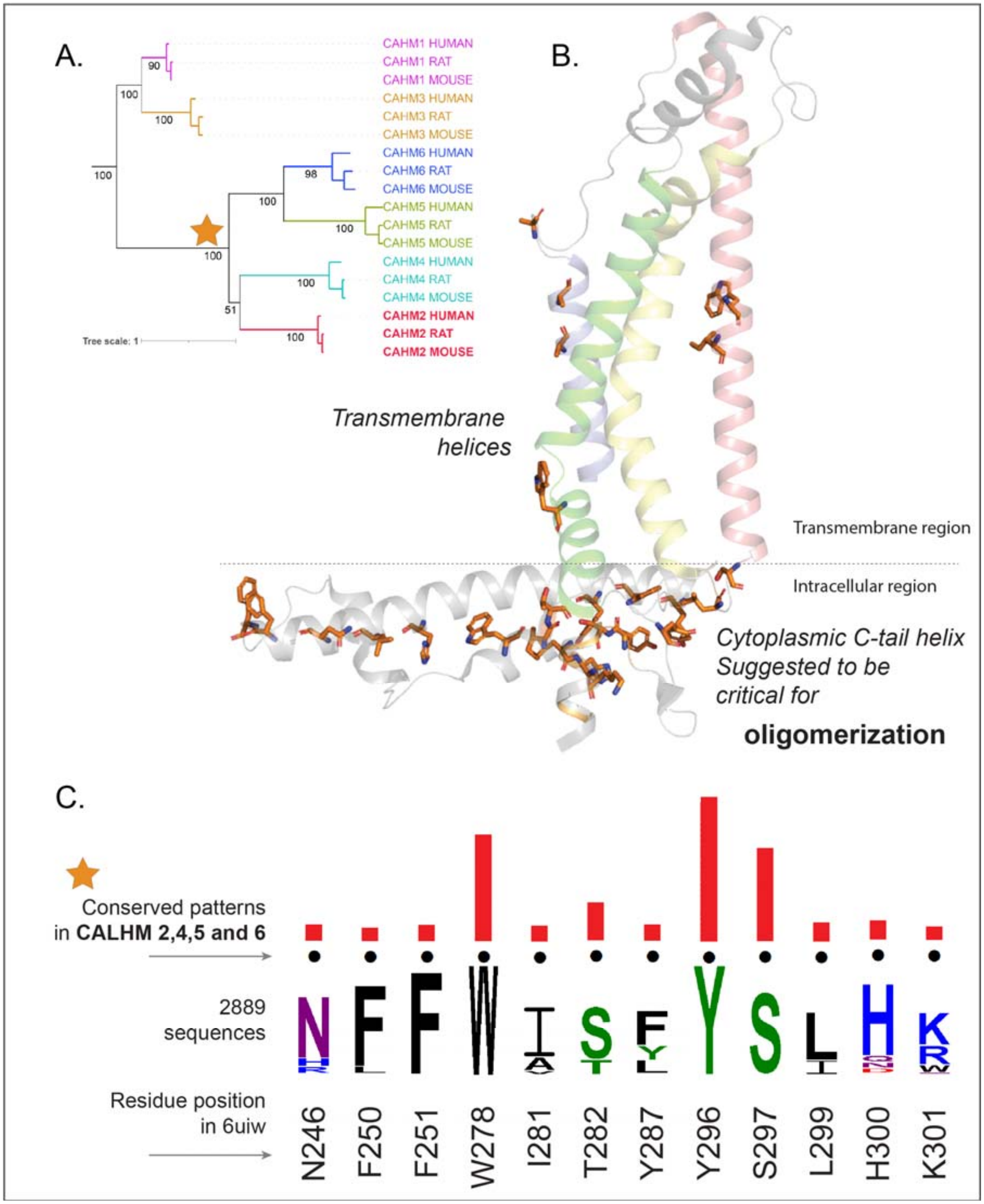

Supplementary Figure 6: Conserved pattern positions identified within the clade for CALHM2,4,5 and 6. A) Phylogenetic tree of CALHM sequences where the orange star indicates the clade for CALHM2,4,5 and 6. B) The identified pattern positions are mapped into a representative structure of human CALHM2 (PDB: 6uiw). C) Weblogo showing the conserved pattern positions. The red bar indicates the significance of conservation where a taller bar indicates higher significance.
